# So-Called Influenzal Attacks Which Are Really Indicative of Phthisis

**Published:** 1910-09-17

**Authors:** 


### MEDICINE.

### SO-CALLED INFLUENZAL ATTACKS WHICH ARE REALLY INDICATIVE OF PHTHISIS.

All observers who have made phthisis a study are agreed that the great majority of cases run their course by a process which may be described as that of exacerbations with intermissions. These exacerbations and intermissions are obvious enough when a stage of the malady has been reached when there is little or no doubt as to the diagnosis, but it is too seldom recognised that long before there are definite abnormal physical signs in the lungs, and in many cases even before there are such definite symptoms as cough or hæmoptysis, the disease produces little outbursts of pyrexia associated with temporary lassitude which may last a few days or a week and then pass off entirely for the time being, exactly like those attacks which have been again and again spoken of as influenza. It is far too easy to diagnose an attack of "influenza," and to consider that that diagnosis is final. Many attacks which are called influenza are really what the French call *pousses évolutives de la tuberculose.* The importance of recognising this is that if there is even the smallest quantity of sputum obtainable in such cases it should be examined carefully for tubercle bacilli as well as for the bacillus of influenza; for if tubercle bacilli can be found in it at an early stage when the patient exhibits no wasting, no night sweats, none of the severe toxic effects of phthisis and secondary infection of cocci, the adoption of suitable precautions may lead to almost immediate cure. It is too late to wait until there are definite physical signs, for phthisis may be diagnosed by microscopical examination for tubercle bacilli in the sputum at a much earlier date than that at which the changes in the lungs have been sufficient to produce definitely abnormal signs. One can hardly say too much in depreciation of the tendency there is to be satisfied with the diagnosis of "influenza" when the only evidence of the malady is a temporary pyrexia.

The *pousses évolutives* of established phthisis are characterised by a marked increase in both the general and the functional symptoms of the malady. Amongst these symptoms the temperature is of particular interest. A rise of temperature is the rule, and only in exceptional cases does the temperature remain normal. It is not a question of transitory rises, such as may be noticed in the evenings in tuberculous subjects after a fatiguing walk, for instance, but of definite cyclical curves; a period of rise, a period of maintained temperature, and a period of defervescence.

### Clinical Types.

The clinical types of these curves are very variable. In certain cases it is almost a matter of ephemeral elevation, or the thermal curve may show a more lasting rise, the temperature going rapidly up to 102° or 103° F., either at one leap or by two or three stages, maintaining these figures for 10 days or a fortnight, and then falling again more or less by lysis. In yet other cases the thermic curve will extend over several weeks, producing a very elongated chart not unlike that of some cases of persistent typhoid fever. Generally speaking, in this last form the temperature rises slowly to a maximum about which it remains for a longer or shorter time, and then falls with extreme slowness, and in certain cases in which defervescence is very pronounced the cyclical curve may not become obvious until reduced charts extending over several months can be obtained. In this form the defervescence takes place so gradually that it may at any given moment seem as though there were no improvement at all, and yet the latter comes about when all hope seems to have been lost. Occasionally a thermic curve at the moment of defervescence presents quite a peculiar aspect like that of a ladder with very accentuated steps. Whatever may be the actual appearance of this pyrexial curve, it presents three main characters almost constantly: it is cyclical, it is of remittent type, and, generally speaking, it does not present a terminal hypothermia.

There may be many different intercurrent factors, especially secondary infection of cavities in the lung by pyogenic cocci, which interfere with the curves; but not only in the earlier stages of phthisis, when there are as yet no physical signs, may there be *pousses évolutives* which are apt to be labelled "influenzal," but also in the later stages of the disease these *pousses évolutives* are often apparent.

The Importance of Examining the Sputum.

Parallel with the temperature characters the arterial blood-pressure shows changes of some interest. At the moment of the *pousses évolutives* the arterial blood-pressure, which is always low in tuberculous subjects, falls still more, and may reach such figures as even 10, 9, or 8 of Potain's sphygmomanometer. The end of the *pousse* is, on the contrary, marked by a slight rise in the blood-pressure up to 12 or even 14, though in the majority of cases it rises only with extreme slowness and always remains below the normal. There is, nevertheless, as in any other infectious malady, a fall of pressure in the first instance, followed by a definite rise at the moment of convalescence.

The patient at the same time exhibits exacerbations in his other symptoms and physical signs. There is often a temporary increase in the sputum. If phthisis has reached a stage at which there is already a more or less abundant expectoration at the moment of the *pousse*, there may be an increase in the quantity of this expectoration without material change in its character; in other cases the sputum becomes more purulent, and often it develops a viscosity which makes it more or less adherent to the side of the vessel into which it is put; sometimes it develops an amber-yellow colour and a slightly translucent aspect which is almost typical, and it may even become so like that of ordinary pneumonia that unless a microscopical examination is made an error of diagnosis may occur although tubercle bacilli are present in large numbers. Sometimes the patient is one who has had no expectoration at all previous to the onset of the *pousse*; one then notices that he begins to bring up a small amount of purulent sputum for a short time, and possibly even only for a single day. It is very easy in such cases to imagine that the expectoration comes from the back of the nose or from the pharynx. The chief feature of the microscopical change in the sputum during a *pousse* is the marked increase in the number of tubercle bacilli it contains. This increase of the latter may be quite ephemeral, but if carefully looked for it is nearly always marked. Quite a number of these patients never spit blood at all; but in a few instances one notices how the hæmoptysis, slight though it is, is definitely related to the *pousses évolutives*. The great danger is that the cause of such a *pousse* may be overlooked, and that it may be mis-diagnosed as "influenza," unless care be taken never to diagnose influenza except when there can be no doubt about it.

